# Exploring Two Decades of Cancer Trends in Adolescents and Young Adults: Insights From a Resource-Restricted Country

**DOI:** 10.14740/wjon2731

**Published:** 2026-05-08

**Authors:** Maha Barbar, Asem Mansour, Rawad Rihani, Iyad Sultan, Sarah Abdel-Razeq, Ronny Baqain, Ayat Taqash, Hira Bani Hani, Hikmat Abdel-Razeq

**Affiliations:** a Department of Pediatrics, King Hussein Cancer Center, Amman 11941, Jordan; b School of Medicine, the University of Jordan, Amman 11941, Jordan; c Department of Radiology, King Hussein Cancer Center, Amman 11941, Jordan; d University of Manchester, Manchester M13 9PL, UK; e Office of Scientific Affairs and Research, King Hussein Cancer Center, Amman 11941, Jordan; f Department of Internal Medicine, King Hussein Cancer Center, Amman 11941, Jordan

**Keywords:** AYA, Jordan, Low-resourced countries, LMIC, Global Oncology

## Abstract

**Background:**

Over 40% of Jordan’s population falls within the adolescent and young adult (AYA) age group (15–39 years). Cancer diagnosed in this population has unique biological, clinical, and psychosocial characteristics. This study describes national incidence trends and treatment outcomes among AYA patients in Jordan.

**Methods:**

This retrospective observational study utilized data from Jordan Cancer Registry (JCR) reports (2000–2022), King Hussein Cancer Center (KHCC) registry outcomes, and the latest GLOBOCAN report. Descriptive statistics, Kaplan–Meier survival analysis, and log-rank testing were used to evaluate cancer incidence and overall survival.

**Results:**

The median age at cancer diagnosis in Jordan is 57 years, significantly younger than in Western countries. Over the past 23 years, the total number of cancer cases among AYA increased from 654 in 2000 to 1,167 in 2022, representing 13.3% of all cancer diagnoses in that year. Most cases (36.8%) occurred in the older AYA subgroup (35–39 years). Age-standardized incidence rates (ASIRs) were consistently higher in females than males across all AYA age groups, largely driven by breast and thyroid cancers. ASIR increased from 17.3 per 100,000 in the youngest group (15–19 years) to 84.4 per 100,000 in the oldest group (35–39 years). The 5-year overall survival (OS) among AYA patients was 73.0% (95% confidence interval (CI), 71.8–74.1), significantly better than older adults at 57.1% (95% CI, 56.4–57.8).

**Conclusion:**

Jordan’s population is predominantly young, with over 40% classified as AYA. Although cancer incidence is lower in this age group compared with older adults, outcomes are generally more favorable. A comprehensive AYA oncology strategy incorporating specialized psychosocial, fertility, and survivorship services should be prioritized, particularly in resource-restricted settings.

## Introduction

Cancer continues to be a major health care challenge worldwide, particularly in resource-restricted countries where disease burden is rapidly rising [1]. The introduction of expensive new anti-cancer therapies, including immunotherapy, targeted therapy, and cellular therapy, along with increased reliance on advanced molecular diagnostics, has further intensified disparities in access to cancer care in low- and middle-income countries (LMICs) [2].

Jordan’s population has grown substantially over the past decade, largely due to the influx of refugees from neighboring countries [3, 4]. This growth has placed considerable strain on an already resource-limited health care system. Jordan’s economy continues to experience slow growth, high unemployment, and limited natural resources. With a gross domestic product (GDP) of approximately USD 50 billion and a GDP per capita below USD 4,500, the World Bank currently classifies Jordan as an LMIC [5].

Over the past decade, adolescents and young adults (AYAs) have increasingly been recognized as a distinct oncology population. However, until recently, there was no universal consensus on the age boundaries defining this group. The World Health Organization (WHO) [6] and Surveillance, Epidemiology, and End Results (SEER) program historically used younger age cutoffs [7]. The age range of 15–39 years, adopted by the National Cancer Institute (NCI) [8] and National Comprehensive Cancer Network (NCCN) [9], is now widely accepted and is used in this study. In Jordan, pediatric age cutoffs vary substantially across institutions, ranging from 14 to 18 years, further underscoring the need for a standardized definition when reporting national AYA cancer data.

Jordan has a young population, with nearly 75% under the age of 40 and approximately 42.2% within the AYA age group (Supplementary Materials 1 and 2, wjon.elmerpub.com). This demographic profile likely contributes to the relatively lower national cancer incidence rates compared with Western countries. However, ongoing demographic transitions toward older age structures are expected to result in substantial increases in cancer burden in the coming decades.

Young patients diagnosed with cancer face unique developmental, social, educational, reproductive, and psychological challenges that differ from those of children or older adults. These challenges necessitate age-specific care models that extend beyond conventional oncology services.

Tumor biology and treatment outcomes also vary by age. Acute lymphoblastic leukemia (ALL), for example, demonstrates markedly different outcomes when treated using pediatric versus adult protocols [10, 11]. Many younger adults with ALL are therefore treated using pediatric-inspired regimens. Overall, survival outcomes among pediatric and AYA patients tend to exceed those observed in older adults [12].

Because most AYA patients survive their cancer, the long-term consequences of treatment, including infertility, metabolic complications, cardiovascular disease, secondary malignancies, and psychosocial sequelae, become critically important [13–15]. Developmental stage, socioeconomic factors, insurance coverage, access to care, and clinical trial participation all influence outcomes and survivorship in this population. Underrepresentation of AYA patients in clinical trials further limits evidence-based advances for this group [16–18].

In this manuscript, we present national data on cancer incidence trends among AYA patients in Jordan and treatment outcomes from the largest cancer center in the country. These data aim to support the establishment of structured AYA oncology services even in resource-restricted settings.

## Materials and Methods

### Study design and data sources

This was a retrospective, population-based observational study evaluating cancer incidence trends and survival outcomes among AYA in Jordan over a 23-year period (2000–2022). Incidence and demographic data were obtained from annual reports published by the Jordan Cancer Registry (JCR), a national, population-based registry that captures cancer diagnoses from all public and private healthcare institutions across the country using standardized reporting procedures and international coding systems [19, 20].

Survival and detailed clinical data were obtained from the institutional cancer registry at King Hussein Cancer Center (KHCC), established in 2006. This registry includes prospectively collected patient-level data on demographics, tumor characteristics, staging, treatment modalities, and outcomes for patients treated at KHCC. To enable international benchmarking and contextualization of national trends, additional population-level cancer incidence data were retrieved from the Global Cancer Observatory (GLOBOCAN) database of the International Agency for Research on Cancer (IARC) [21].

### Study population

All newly diagnosed malignant neoplasms reported to the JCR between January 1, 2000, and December 31, 2022, in individuals aged 15–39 years were included and classified as AYA cancers. Patients younger than 15 years were categorized as pediatric cases, and those aged 40 years or older as older adults, in accordance with international age-group definitions.

For survival analyses, the study cohort was restricted to patients diagnosed and treated at KHCC with available follow-up data. Patients with incomplete survival information or without confirmed histopathological diagnoses were excluded from survival analyses but retained in incidence analyses when applicable. Cancers were grouped according to major diagnostic categories based on the International Classification of Diseases for Oncology (ICD-O) and standard AYA cancer classification frameworks.

### Variables and outcomes

Collected variables included age at diagnosis, sex, year of diagnosis, cancer site and histology, and stage at presentation when available. The primary outcome was overall survival (OS), defined as the time from histologically confirmed primary cancer diagnosis to death from any cause or last documented follow-up. Patients who were alive at last contact were censored at that date.

### Statistical analysis

Descriptive statistics were used to summarize demographic and clinical characteristics. Categorical variables were reported as frequencies and percentages, while continuous variables were summarized using medians and interquartile ranges (IQRs). Temporal trends in cancer incidence were examined descriptively across calendar years and age groups.

OS was estimated using the Kaplan–Meier method, and survival curves were compared across age groups using the log-rank test. Survival estimates were reported with corresponding 95% confidence intervals (CIs). A two-sided P-value ≤ 0.05 was considered statistically significant. All statistical analyses were conducted using standard statistical software packages.

### Ethical statements

The study was approved by the Institutional Review Board at KHCC. As this study used de-identified registry-based data with no direct patient contact, the requirement for informed consent was waived in accordance with institutional and national regulations.

## Results

### National cancer epidemiology (all ages)

In 2022, the JCR reported 8,754 new cancer cases nationwide, including 4,736 (54.1%) among females. The median age at diagnosis was 57 years (females: 54 years; males: 60 years). The overall crude incidence rate was 111.9 per 100,000, with higher rates in females (123.3) than males (100.8). The age-standardized incidence rate (ASIR), adjusted to the world standard population, was 157.4 per 100,000.

**Figure 1:**
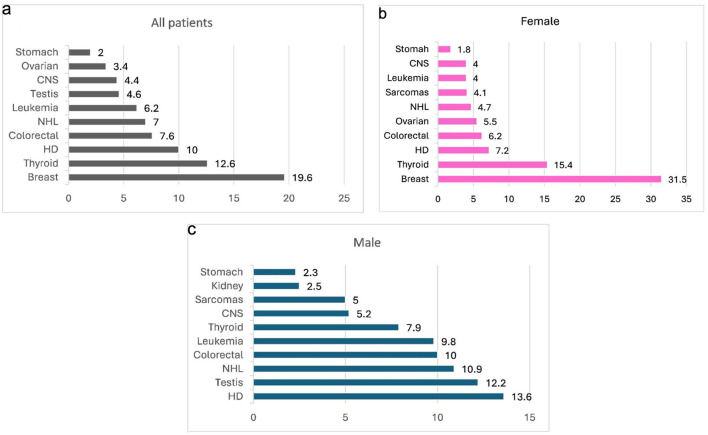
Most common cancers among AYA patients (%), year 2000–2022. (a) Distribution of the most common cancers reported across the entire AYA population. (b) Most common cancers among female AYA patients. (c) Most common cancers among male AYA patients. AYA: adolescent and young adult; CNS: central nervous system; HD: Hodgkin lymphoma; NHL: non-Hodgkin lymphoma.

Only 312 cases (3.6%) occurred in children under 15 years of age. Breast cancer (20.1%), colorectal cancer (11.1%), lung cancer (7.4%), lymphoma (7.0%), and bladder cancer (5.4%) were the five most common cancers across both sexes and all AYA age groups (Fig. 1). Lung cancer was the leading cause of cancer-related death among males (22.4%), while breast cancer was the leading cause among females (24.9%).

### AYA cancer incidence trends

**Figure 2:**
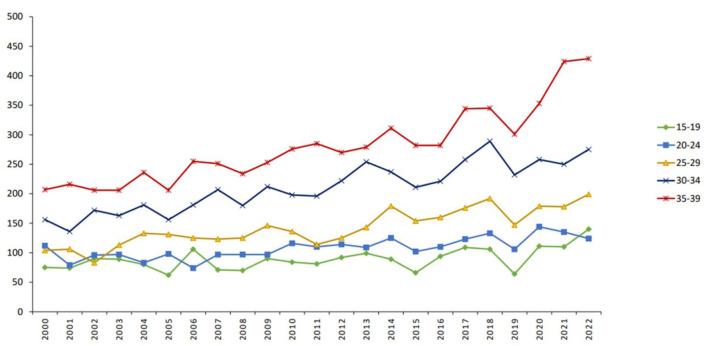
Trends in AYA cancer incidence across age group categories (number). AYA: adolescent and young adult.

In 2022, 1,167 new cancer cases were diagnosed among individuals aged 15–39 years, representing 13.3% of all cancers diagnosed nationwide. Over one-third of these cases (36.8%) occurred in the older AYA subgroup (35–39 years), followed by 23.6% in those aged 30–34 years, with progressively lower incidence in younger age strata. Figure 2 illustrates the annual increase in AYA cancer diagnoses over the 23-year study period by age subgroup.

Over the past two decades, the total number of AYA cancer cases increased from 654 in 2000 to 1,167 in 2022. Among males, Hodgkin lymphoma (13.6%), testicular cancer (12.2%), non-Hodgkin lymphoma (10.9%), colorectal cancer (10.0%), and acute leukemia (9.8%) were the most common malignancies. Among females, breast cancer predominated (31.5%), followed by thyroid cancer (15.4%), Hodgkin lymphoma (7.2%), colorectal cancer (6.2%), and ovarian cancer (5.5%) (Fig. 1). In children under 15 years, acute leukemia (24.4%) and central nervous system tumors (20.2%) accounted for nearly half of all malignancies.

### ASIRs by sex and age

**Figure 3:**
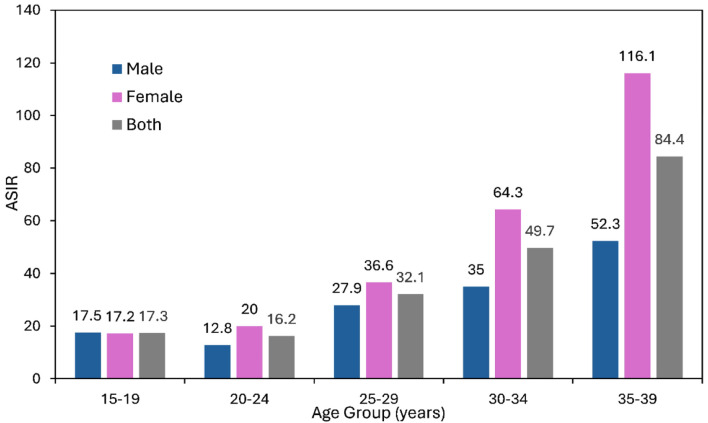
Age-standardized incidence rate (ASIR) of AYA cancers by age group (per 100,000 population), year 2000–2022. AYA: adolescent and young adult.

The latest JCR data demonstrate consistently higher ASIRs among females than males across all AYA age subgroups. This difference was most pronounced in older age groups: in individuals aged 30–34 years, the ASIR was 64.3 per 100,000 for females versus 35.0 per 100,000 for males, and in those aged 35–39 years, 116.1 versus 52.3 per 100,000, respectively. These disparities were largely attributable to breast cancer and, to a lesser extent, thyroid cancer (Fig. 3).

ASIRs increased progressively with age within the AYA population, rising from 17.3 per 100,000 in the 15–19-year age group to 49.7 per 100,000 in those aged 30–34 years and reaching 84.4 per 100,000 in those aged 35–39 years.

### Survival outcomes

**Figure 4:**
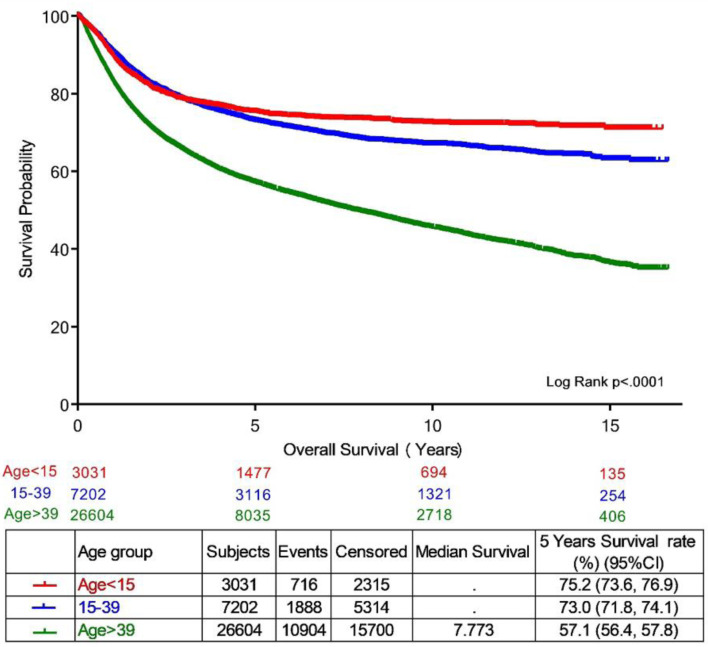
Overall survival of AYA cancer patients by age group category, year 2000–2022. AYA: adolescent and young adult.

Survival data were available for 7,202 AYA patients treated and followed at KHCC. The 5-year OS was 73.0% (95% CI, 71.8–74.1), significantly higher than the 57.1% (95% CI, 56.4–57.8) observed among 26,604 older adults (≥ 40 years) and slightly lower than the 75.2% (95% CI, 73.6–76.9%) among 3,031 pediatric patients (< 15 years) treated during the same period (P < 0.0001; Fig. 4). These findings highlight the intermediate survival outcomes of AYA patients between pediatric and older adult populations.

## Discussion

Jordan has a young population, with a median age of 24.5 years, making cancer among AYAs a particularly important public health concern. This study provides the most comprehensive longitudinal analysis of AYA cancer incidence and outcomes in Jordan and one of the largest reports from an LMIC.

Consistent with international experience, AYA patients demonstrated higher survival rates than older adults, but slightly inferior outcomes compared with pediatric patients. This pattern likely reflects age-related differences in tumor biology, treatment tolerance, treatment intensity, and comorbidity burden. Tumor spectrum also varies substantially across age groups, with lymphomas, leukemias, germ cell tumors, and breast cancer predominating in the AYA population.

Although most AYA patients survive their cancer, they face substantial physical, psychological, social, and economic challenges that extend well beyond treatment completion (Table 1). Late effects, defined as adverse outcomes occurring ≥ 5 years after treatment, remain underrecognized in LMICs despite their significant long-term burden. The Childhood Cancer Survivor Study (CCSS) has provided critical insights into these outcomes [22]. In a cohort of 5,804 early-AYA cancer survivors (aged 15–20 years at diagnosis), the standardized mortality ratio (SMR) for all-cause mortality was 5.9 (95% CI, 5.5–6.2) compared with the general population, similar to the SMR of 6.2 (95% CI, 5.8–6.6) observed among childhood cancer survivors [23]. These data underscore the need for structured survivorship care models tailored to AYA populations.

**Table 1:** Challenges in AYA Cancer Management

Key issues	Clinical and supportive needs	Barriers and challenges
Availability of early detection and screening tools (when indicated)	Need for advanced imaging and screening modalities beyond routine recommendations (e.g., breast MRI instead of mammogram).	Financial burden/high cost; limited convenience; restricted access
More aggressive disease compared to older patients	Requires intensified treatment approaches, including chemotherapy and immunotherapy (e.g., higher prevalence of triple-negative breast cancer).	Financial burden/high cost; delayed adverse events; limited access and availability of new drugs
Higher prevalence of pathogenic/likely pathogenic (P/LP) germline mutations	Need for standardized, simplified genetic testing guidelines across all tumor types.	Financial burden/high cost; family communication challenges and the complexity of cascade testing; risk of stigmatization
Fertility preservation	Many chemotherapeutic agents may impair fertility. Oocyte preservation and sperm banking should be addressed with patients after proper counseling. The process can be particularly challenging for younger patients.	Financial burden/high cost; limited access to fertility preservation services; procedural difficulty, especially for younger patients
Body image concerns	Need for access to reconstructive and plastic surgery (e.g., mastectomy reconstruction). Psychological impact on body image.	High cost of procedures; limited availability of specialized surgical services

AYA: adolescent and young adult.

AYA cancer patients frequently experience disruptions in education, employment, social development, and psychosocial well-being [24, 25]. Emotional distress, anxiety, depression, and impaired quality of life are common, particularly during active treatment and early survivorship [26, 27]. These vulnerabilities necessitate dedicated psychological and social support services embedded within AYA oncology programs.

Fertility preservation represents a particularly critical issue for AYA patients. Many anti-cancer therapies adversely affect gonadal function in both sexes. Amenorrhea may occur in over half of young women receiving combination chemotherapy [28]. Fertility preservation strategies include sperm banking, oocyte or embryo cryopreservation, ovarian tissue cryopreservation, and the use of gonadotropin-releasing hormone agonists (GnRHa) during chemotherapy [29–32]. Despite available interventions, fertility counseling and preservation services remain underutilized in many LMICs, underscoring the need for systematic integration into routine cancer care.

Metabolic and endocrine complications, particularly obesity, represent another major survivorship challenge. The prevalence of obesity among childhood and AYA cancer survivors ranges from 30% to 50% in various cohorts [33–37], especially among survivors of ALL and brain tumors [38–41]. Obesity contributes to premature cardiovascular disease, endocrine dysfunction, and reduced quality of life. Survivorship from childhood cancer is recognized by the American Heart Association as an independent cardiovascular risk factor [42, 43]. Therefore, early metabolic screening and lifestyle interventions are essential components of survivorship care.

Bone health is also adversely affected by chemotherapy, corticosteroids, endocrine therapies, and premature gonadal failure [44–47]. Osteopenia and osteoporosis may occur, particularly in patients exposed to cranial irradiation or prolonged steroid therapy. Preventive strategies include exercise, vitamin D supplementation, calcium intake, and, in selected cases, pharmacologic therapy with bisphosphonates or denosumab [48].

### Study limitations

Our study has several limitations. The retrospective design and use of survival data from a single tertiary cancer center may limit national generalizability. However, KHCC treats the majority of cancer patients in Jordan, particularly within the AYA population, and maintains a mature and comprehensive institutional registry. Additionally, national survival data are not currently available, making institutional registries the most reliable source for outcome analyses. Nevertheless, our large sample size and extended follow-up period strengthened the robustness of our findings.

### Conclusions

Nearly 75% of Jordan’s population is under the age of 40, and over 40% falls within the AYA age group. Although cancer incidence is lower in this population compared with older adults, outcomes are generally favorable, and survivorship issues are substantial. Establishing comprehensive AYA oncology programs incorporating fertility preservation, psychosocial support, lifestyle interventions, and structured survivorship care should be prioritized, even in resource-restricted settings.

## Supplementary Material

Suppl 1 Population pyramid of Jordan.

Suppl 2 Population of Jordan by gender and age groups – 2023 and projected population of Jordan by gender and age groups – 2050.

## Data Availability

The data supporting the findings of this study are available from the corresponding author upon reasonable request.
