# Supplementary figures and images for: Exploring Two Decades of Cancer Trends in Adolescents and Young Adults: Insights From a Resource-Restricted Country
Source: World J Oncol. 2026 May 8;17(3):357–65. doi: 10.14740/wjon2731 (PMC13171271; doi:10.14740/wjon2731)

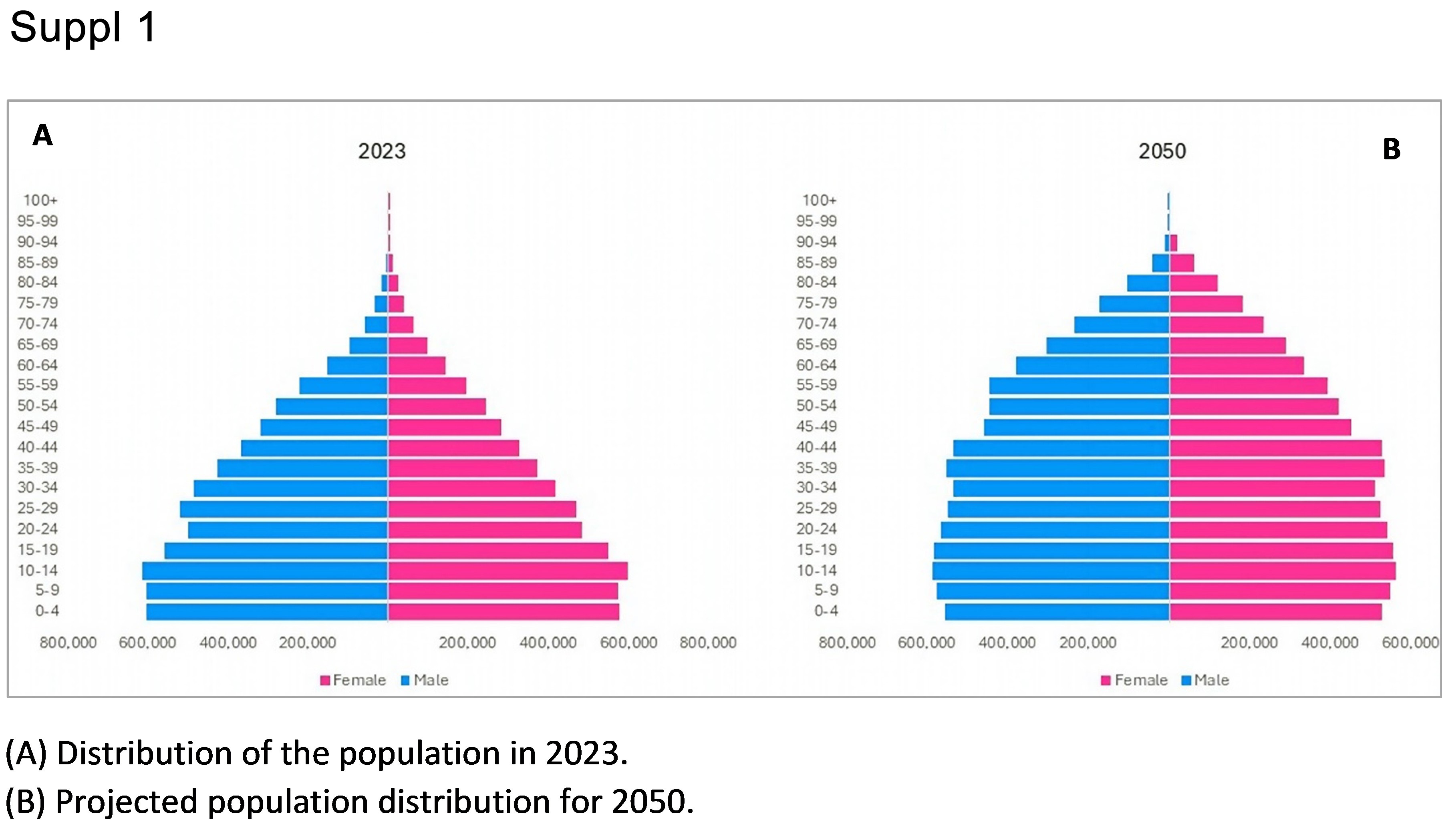

Supplement: Suppl 1 — Population pyramid of Jordan. [file wjon-17-03-357-s001.jpg]
