# Supplementary material for: Exploring Two Decades of Cancer Trends in Adolescents and Young Adults: Insights From a Resource-Restricted Country
Source: World J Oncol. 2026 May 8;17(3):357–65. doi: 10.14740/wjon2731 (PMC13171271; doi:10.14740/wjon2731)
Supplement: Suppl 2 — Population of Jordan by gender and age groups – 2023 and projected population of Jordan by gender and age groups – 2050. [file wjon-17-03-357-s002.docx]

**Suppl 2.**

**Population of Jordan by gender and age groups - 2023**

| **Age Group** | **Male** | **Female** |
| --- | --- | --- |
| 0-4 | 594793 | 568477 |
| 5-9 | 607350 | 580301 |
| 10-14 | 616485 | 604626 |
| 15-19 | 554994 | 552045 |
| 20-24 | 498798 | 487502 |
| 25-29 | 523398 | 480469 |
| 30-34 | 491998 | 426436 |
| 35-39 | 425594 | 375815 |
| 40-44 | 364054 | 328402 |
| 45-49 | 317802 | 283889 |
| 50-54 | 283809 | 247883 |
| 55-59 | 226308 | 199809 |
| 60-64 | 157435 | 146295 |
| 65-69 | 103729 | 101187 |
| 70-74 | 68662 | 67940 |
| 75-79 | 38959 | 43137 |
| 80-84 | 20162 | 26154 |
| 85-89 | 7396 | 11909 |
| 90-94 | 1400 | 3192 |
| 95-99 | 117 | 465 |
| 100+ | 3 | 23 |

**Projected population of Jordan by gender and age groups - 2050**

| **Age** | **Males** | **Females** |
| --- | --- | --- |
| 0-4 | 623330 | 594957 |
| 5-9 | 644510 | 615766 |
| 10-14 | 640401 | 612392 |
| 15-19 | 619259 | 592649 |
| 20-24 | 624883 | 597600 |
| 25-29 | 626081 | 601155 |
| 30-34 | 618436 | 595200 |
| 35-39 | 629454 | 611790 |
| 40-44 | 581431 | 585431 |
| 45-49 | 466842 | 467747 |
| 50-54 | 430472 | 415711 |
| 55-59 | 449040 | 401577 |
| 60-64 | 392129 | 350540 |
| 65-69 | 323936 | 307786 |
| 70-74 | 257196 | 253977 |
| 75-79 | 196597 | 201620 |
| 80-84 | 124132 | 137468 |
| 85-89 | 54373 | 71681 |
| 90-94 | 14836 | 25879 |
| 95-99 | 2236 | 5926 |
| 100+ | 155 | 764 |
